# Extended passaging increases the efficiency of neural differentiation from induced pluripotent stem cells

**DOI:** 10.1186/1471-2202-12-82

**Published:** 2011-08-10

**Authors:** Karl R Koehler, Philippe Tropel, Jonathan W Theile, Takako Kondo, Theodore R Cummins, Stéphane Viville, Eri Hashino

**Affiliations:** 1 Stark Neurosciences Research Institute; 2 Department of Otolaryngology; 3 Department of Pharmacology and Toxicology, Indiana University School of Medicine, Indianapolis, IN 46202, USA; 4 Service de Biologie de la Reproduction, Centre Hospitalier Universitaire, Strasbourg, F-67000 France; 5 Institut de Génétique et de Biologie Moléculaire et Cellulaire (IGBMC), Institut National de Santé et de Recherche Médicale (INSERM) U964/Centre National de Recherche Scientifique (CNRS) UMR 1704/Université de Strasbourg, 67404 Illkirch, France

## Abstract

**Background:**

The use of induced pluripotent stem cells (iPSCs) for the functional replacement of damaged neurons and *in vitro *disease modeling is of great clinical relevance. Unfortunately, the capacity of iPSC lines to differentiate into neurons is highly variable, prompting the need for a reliable means of assessing the differentiation capacity of newly derived iPSC cell lines. Extended passaging is emerging as a method of ensuring faithful reprogramming. We adapted an established and efficient embryonic stem cell (ESC) neural induction protocol to test whether iPSCs (1) have the competence to give rise to functional neurons with similar efficiency as ESCs and (2) whether the extent of neural differentiation could be altered or enhanced by increased passaging.

**Results:**

Our gene expression and morphological analyses revealed that neural conversion was temporally delayed in iPSC lines and some iPSC lines did not properly form embryoid bodies during the first stage of differentiation. Notably, these deficits were corrected by continual passaging in an iPSC clone. iPSCs with greater than 20 passages (late-passage iPSCs) expressed higher expression levels of pluripotency markers and formed larger embryoid bodies than iPSCs with fewer than 10 passages (early-passage iPSCs). Moreover, late-passage iPSCs started to express neural marker genes sooner than early-passage iPSCs after the initiation of neural induction. Furthermore, late-passage iPSC-derived neurons exhibited notably greater excitability and larger voltage-gated currents than early-passage iPSC-derived neurons, although these cells were morphologically indistinguishable.

**Conclusions:**

These findings strongly suggest that the efficiency neuronal conversion depends on the complete reprogramming of iPSCs via extensive passaging.

## Background

Induced pluripotent stem cells (iPSCs) are somatic cells that have been epigenetically reprogrammed to a pluripotent state using the ectopic expression of defined factors (Oct3/4, Sox2, Klf4, c-myc, Nanog or Lin28) or small molecule treatments [1-5]. Like embryonic stem cells (ESCs), iPSCs have the ability to differentiate into all three germ layers and thus, represent a viable option for autologous cell replacement therapies. A number of groups have investigated the potential of iPSCs for generating *in vitro *models of neurodegenerative maladies, such as, Parkinson's disease, retinal degeneration, amyotrophic lateral sclerosis and Rett Syndrome [6-14]. Although these studies are encouraging, little is currently known about the molecular underpinnings of reprogramming and the faithfulness with which iPSCs can recapitulate neuronal differentiation.

Although iPSCs of both mouse and human origins appear morphologically indistinguishable from ESCs, several reports have emerged showing variations at the transcriptomic and epigenomic levels [15-22]. In contrast, studies by Guenther et al. [23] and Neumann and Cooper [24], have shown convincingly that the discrepancies between iPSCs and ESCs are not significantly different from variations between ESC lines with divergent genetic backgrounds [23]. Moreover, laboratory-specific factors such as culture conditions and reprogramming methods may be an underlying cause of these observed differences [24]. Variations in teratoma forming ability, hematopoiesis and neuronal differentiation have been observed among mouse and human iPSC lines [25]. Recently, Polo et al. [26], Kim et al. [27] and Marchetto et al. [28], observed that many early-passage mouse iPSC lines maintain a persistent epigenetic signature of the tissue type of origin. Interestingly, when directed to differentiate to hematopoietic or osteogenic cell types, these early-passage cells were biased toward their original cell state, thus leading to low differentiation efficiency [26,27]. At later passages, the iPSCs differentiated more efficiently, which led the researchers to conclude that a period of prolonged cellular proliferation may be a necessary component of the reprogramming process. In light of these findings, it has become clear that newly derived iPSC lines should be thoroughly characterized based on their expression of endogenous pluripotency genes, morphology and differentiation capacity. However, information is lacking whether extensive passaging has effects on the competence of iPSCs to give rise efficiently to a neuronal lineage.

The goal of this study was to assess the effects of passaging on genetic stability in iPSCs and their efficiency in giving rise to functional neurons. We also wished to compare the neural differentiation potential of iPSCs with that of ESCs, by performing quantitative evaluation of temporal expression patterns of a battery of genes expressed sequentially during neural development. Due to the reported similarities between iPSC and ESCs, we hypothesized that both cells undergo similar transitions in the expression of key markers of neural differentiation. We found that iPSC lines we generated had variable competence to generate neural cells. We speculated that these discrepancies could stem from the inherent heterogeneity of iPSC cultures prior to differentiation or a residual epigenetic signature from the tissue of origin [26,27]. We found that, after continual passaging, an iPSC line with a low efficiency of neural conversion could recapitulate the gene expression patterns seen in ESCs undergoing neural differentiation. These findings highlight the importance of extensive cellular turnover for establishing a fully reprogrammed state in iPSCs prior to directed neural differentiation.

## Results

### Newly derived mouse iPSCs show variable neural inductive ability at early-passages

**Figure 1:**
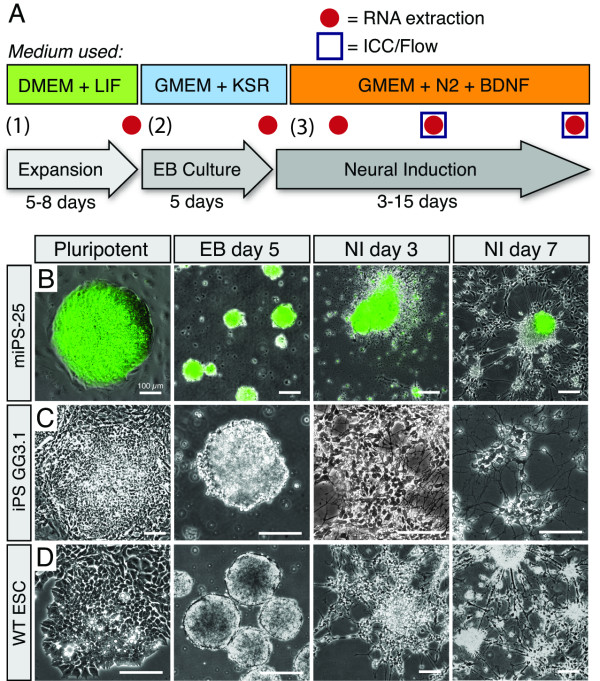
**iPSC lines subjected to neural induction (Ni) produce populations of neurons with similar morphology to ESC-derived neurons**. (**A**) Ni proceeds in three stages: (1) expansion of undifferentiated cells, (2) formation of embryoid bodies (EB) in a floating serum-free culture and (3) neuronal differentiation on poly-d-lysine/laminin coated plates in the presence of N2-supplement and brain derived neurotrophic factor (BDNF). (**B, C**) Representative micrographs for the miPS-25 (**B**), GG3.1 (**C**) and wild-type (WT) ESC lines at critical time points of neural induction: Undifferentiated, EB day 5 and Ni days 3 and 7. Bars represent 100 μm.

We used 4 newly established mouse iPSC lines (denoted as GG3.1/3 and miPS-20/25) and an established ESC line derived from the inner cell mass of an R1 mouse embryo (Additional file 1, Table S1) [29]. Three of the iPSC lines were generated via retroviral transduction of mouse embryonic fibroblasts with mouse *Oct4*, *Sox2*, *Klf4 *(miPS-20) and *Nanog *(miPS-25), whereas the GG3 clones were transduced with human *Oct4*, *Sox2 *and *Klf4*. Notably, the reprogramming factor *c-myc *was omitted to minimize the number of transgenes. The miPS-20/25 lines were generated using fibroblasts from transgenic mice carrying a green florescent protein (GFP) gene driven by the *Oct4 *promoter; therefore, pluripotency and differentiation could be monitored by the expression of GFP (Figure 1B and Additional file 1, Fig. S1A) [30]. iPSCs and ESCs were maintained and subjected to a 2-step neural induction protocol (Figure 1A) as previously described [29]. All cell lines maintained a stereotypical ESC morphology (e.g. enlarged nucleus with prominent nucleoli and rounded cell clusters) in the presence of Leukemia inhibitory factor (LIF) and fetal bovine serum (Figure 1 and Additional file 1, Fig. S1). After adaptation to feeder-free conditions iPSC cultures displayed spontaneous differentiation at the edges of most cell clusters (Figure 1B-D and Additional file 1, Fig. S1). By contrast, spontaneous differentiation in ESC cultures was undetectable.

Pluripotent cells located in the center of these clusters were revealed by alkaline phosphatase staining (Additional file 1, Fig. S2A), which was consistent with GFP expression in miPS-20/25 (Figure 1B and Additional file 1, Fig. S1A). Upon dissociation and placement in serum-free cellular suspension, all cell lines formed embryoid bodies (EB), although the abundance of EBs varied greatly in iPSC cultures (data not shown). When plated and treated with neural induction medium, both ESC and iPSCs displayed characteristic neuronal epithelial morphology within 3 days (i.e. neural tube-like rosettes, Figure 1 and Additional file 1, Fig. S1; Ni3). Neurite-like processes extended from the cell clusters as early as 3 days after the start of neural induction (Figure 1B-D and Additional file 1, Fig. S1). By day 7, neuron-like cells with characteristic bipolar, multipolar and pyramidal morphologies were observed in both ESC and iPSC cultures (Figure 1B-D and Additional file 1, Fig. S1; Ni7). The prevalence of EBs with at least some non-neuronal morphologies was greater than 90% in all early-passage iPSC cultures (n = 3). Specifically, rhythmically beating cells with morphology resembling cardiomyocytes were observed in approximately 10% of plated iPSC EBs and multi-lineage cells were ubiquitous (Additional file 1, Fig. S1C-E, n = 3).

Originally, we had concerns that transgene re-expression may be a confounding factor during the differentiation process due to previous reports of this phenomenon in iPSCs derived using retroviruses [3,4]. However, analysis of endogenous transcripts for the reprogramming factors, *Oct4*, *Sox2 *and *Klf4*, discounted transgene expression in the GG3.1 line (Additional file 1, Fig. S2B). The overall quality of this cell line was further ensured by expression analyses of genes in the *Dlk1-Dio3 *locus on chromosome 12 (Additional file 1, Fig. S2C and D). Recent reports concluded that repression of this locus, specifically the genes *Gtl2 *and *Rian*, is a defining feature of poor quality mouse iPSCs that lack the ability to generate "all-iPSC mice" via tetraploid complementation [31,32]. We analyzed the expression level of *Gtl2 *and *Rian *in the GG3.1 line and found no difference in their expression levels when compared to ESCs (Additional file 1, Fig. S2C and D). Moreover, no significant difference in expression levels of *Gtl2 *and *Rian *was observed between early- and late-passage iPSCs (Data not shown). Considering the final differentiation performance of the GG3.1 line (i.e. post-extended passaging), this method of iPSC quality assessment should prove useful in future experiments where new iPSCs are derived.

**Figure 2:**
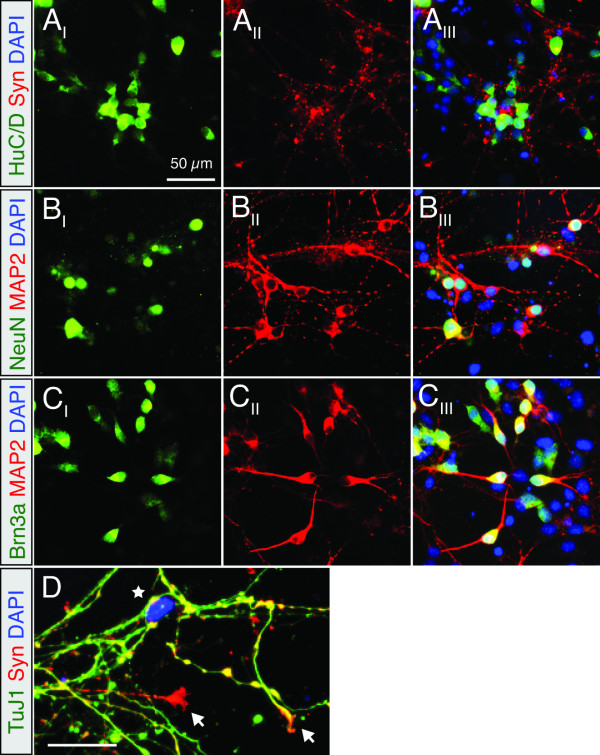
**Neurons derived from GG3.1 iPSCs exhibit characteristic neuronal morphologies and are immuno-positive for several neural markers**. (**A-C**) Representative images of Ni day 7 cells, fixed and stained for (**A**) HuC/D and Synaptophysin (Syn), (**B**) neural nuclei (NeuN) and Map2, or (**C**) Brn3a and Map2. Nuclei are stained with DAPI. (**D**) TuJ1 and Synaptophysin staining reveals the presence of growth cones (arrows) and presumptive synaptic boutons (star) in late-passage GG3.1 cultures on Ni day 7, indicating functional maturity. Bar indicates 50 μm.

To better characterize cellular phenotype, we performed immunocytochemistry on GG3.1 cells at neural induction day 7. Thirty to forty percent (n = 3) of cells stained positive for the early neural marker HuC/D, as well as, the mature neural markers Synaptophysin (Syn), ßIII-tubulin (TuJ1), microtubule associated protein 2 (MAP2) and neural nuclei protein (NeuN). As shown in previous studies, a subset of cells expressed brain-specific homeobox/POU domain protein 3A (Brn3a), indicating the presence of sensory-like neurons (Figure 2A-D). The majority of these cells were also positive for neurofilament and calretinin, consistent with our previous analysis of ESC-derived neurons (Additional file 1, Fig. S3D-F) [29]. Furthermore, we found that Map2, TuJ1, NeuN and neurofilament expression persisted beyond day 15 in iPSC cultures (data not shown). The presence of Syn^+ ^puncta and growth cones was indicative of maturing neurons (Figure 2D). This staining profile is consistent with the forebrain-like neurons observed in our and others' previous ESC analysis (See Additional file 1, Fig. S3A-F for further characterization) [29,33]. From this point on, the GG3.1 and miPS-25 lines were chosen for further analysis based on their disparate methods of generation and ability to form spherical EBs with similar abundance (~0.7-1 × 10^3^/mL, n = 3) as ESCs.

### Extended passaging enhances pluripotent gene expression in an undifferentiated state and increases the rate/efficiency of neuronal conversion

**Figure 3:**
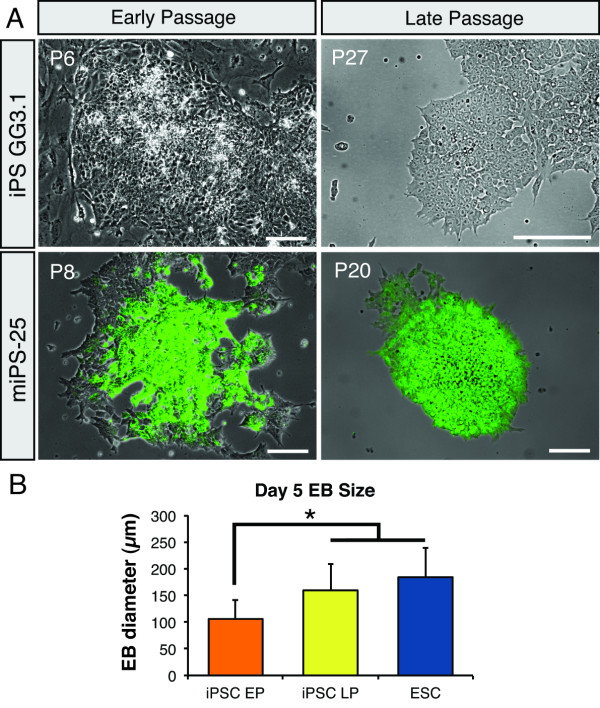
**The effects of continual passaging on cellular morphology, colony shape and EB formation**. (**A**) > 95% of colonies displayed spontaneous differentiation and loss of GFP expression in peripherally located cells of low-passage GG3.1 and miPS-25 cell clusters. Serial passaging results in morphological stability and uniform GFP expression in > 85% of cell colonies in miPS-25 cultures. (**B**) EB diameter increased after multiple passages (iPSC LP represents cells at P20-30) compared to early-passage cultures (iPSC EP represents cells at P7-9). ESC-derived EB diameter also differs significantly from EP-derived EB (n = 3 for each group). Scale bars indicate 100 μm. Values are mean ± SD. *P < 0.01.

**Figure 4:**
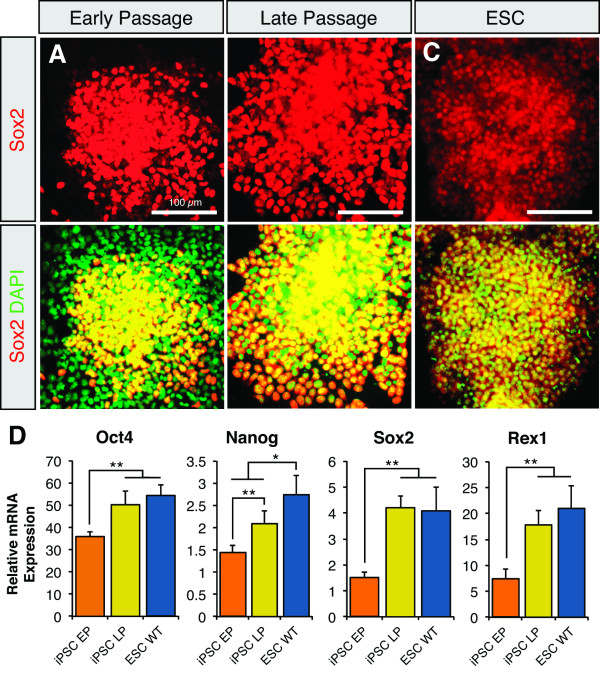
**Late-passage GG3.1 cells are more homogenously undifferentiated and have higher levels of pluripotency factor expression**. (**A**) Comparison of Sox2 stained cells in (**A**) EP, (**B**) LP and (**C**) ESC cultures. LP and ESCs form tight clusters of uniformly Sox2^+ ^cells. Under the same conditions, EP cells readily generate Sox2^- ^cells as indicated by DAPI. (**D**) Real-time qRT-PCR reveals that mRNA expression for several pluripotency factor genes is elevated towards ESC levels in LP GG3.1 cells. Nanog expression remains significantly lower. Values are mean ± SD for 2-3 independent samples. Bars indicate 100 μm. *P < 0.05, **P < 0.001.

Although iPSCs exhibit neural phenotypes similar to ESCs at early-passages, we postulated that the observed morphological and differentiation inconsistencies were a result of either incomplete reprogramming or the heterogeneity of our iPSC cultures. Recent literature suggests that a prolonged period of proliferation and self-renewal may be necessary to stabilize iPSCs in a pluripotent state [17,26]. Accordingly, we passaged iPSCs at least 10 times prior to repetition of neural induction [26]. At 20-30 passages, spontaneous differentiation was undetectable in both GG3.1 and miPS-25 cell lines, whereas GFP expression was uniform in the miPS-25 line (Figure 3A). Interestingly, we observed a significant increase in the diameter of EBs (~90-120 μm up to ~160-190 μm, n = 3) derived from late-passage GG3.1 cells, which was equivalent to the EB size seen in ESC cultures (Figure 3B). Furthermore, relative to early-passage iPSCs, most cells in late-passage GG3.1 cultures expressed Sox2, with few observable differentiated Sox2^- ^cells (Figure 4A and 4B). Real-time qRT-PCR revealed that expression levels of the pluripotency markers *Oct4*, *Sox2*, *Rex1 *and *Nanog *in late-passage cultures were significantly higher than those in early-passage iPSCs and were equivalent to expression levels in ESCs (Figure 4D). Notably, *Nanog *expression in late-passage cells remained significantly lower than in ESCs, but there was an upward trend (Figure 4D).

**Figure 5:**
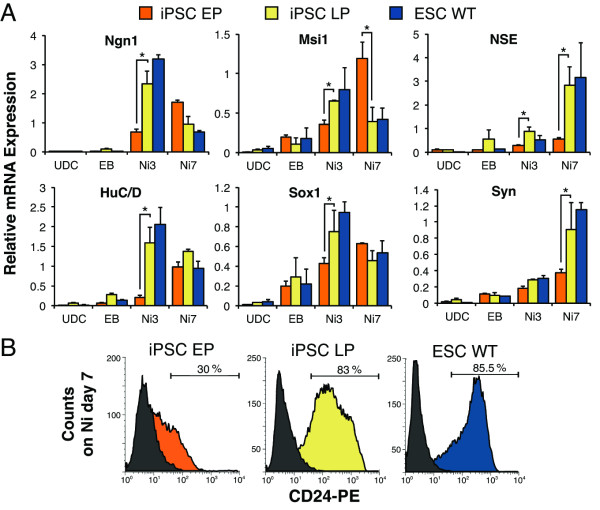
**The temporal pattern of proneural gene expression is equivalent in late-passage GG3.1 iPSCs and ESCs, but delayed or suppressed in early-passage cells**. (**A**) The relative mRNA expression of 4 early markers of neural commitment, Ngn1, Msi1, HuC/D and Sox1, and 2 late markers, NSE and Syn, were quantified for undifferentiated, EB and Ni days 3 and 7 cultures. LP cultures showed a significant increase and temporal shift in the expression of most markers, in congruence with ESC expression patterns. (**B**) The percentage of neural lineage cells in undifferentiated and Ni day 7 EP, LP and ESC cultures as indicated by CD24 cell surface expression. Histograms are representative results of 3 separate experiments are shown. Values are mean ± SD for 2-3 independent samples. *P < 0.05.

To assess the transcriptional changes occurring in iPSCs over the course of neural differentiation, we carried out additional qRT-PCR using cDNA generated from undifferentiated cells, cells at EB day 5, and neural induction days 3 (Ni3), and 7 (Ni7). To clearly delineate events of gene up- and down-regulation, we evaluated the expression of immature- and mature-neuronal markers. Expression of pluripotency markers (*Rex1*, *Oct4 *and *Klf4*) in iPSCs declined promptly during the EB stage and subsequent differentiation (Additional file 1, Figs. S2A and S3B). The immature-neural markers, *Neurogenin1 *(*Ngn1*), *Musashi1 *(*Msi1*), *Sox1 *and *HuC/D *are all transiently expressed during *in vivo *neural development and have been detected in our cultures previously [29,34]. As expected, the mRNA levels of these genes in ESC cultures elevated during early differentiation (Ni3), but declined as neural induction proceeded (Ni7) (Figure 5A). By contrast, the induction of immature-neural marker genes was delayed in early-passage iPSCs (Figure 5A). However, after 20-30 passages, temporal expression patterns and levels of immature-neural markers were not significantly different from ESCs (Figure 5A). We next evaluated the expression of mature neural markers, *neuron specific enolase *(*NSE*), *Syn *(Figure 5A)*, Calretinin *and *TrkB *(Additional file 1, Fig. S2B). We found consistently that expression of these genes is induced by Ni3, but increases dramatically by Ni7 in ESC cultures (Figure 5A). This pattern of expression was seen in early-passage iPSCs, but was not as robust. As with the other markers, late-passage iPSC-derived cultures exhibited significantly higher levels of *NSE* and *Syn* expression than early-passage iPSCs at Ni7 (Figure 5A).

To better quantify the efficiency of neural differentiation, we performed flow cytometry analysis for the neural lineage marker CD24 [35-37]. Our data revealed a lower percentage of CD24^+ ^cells in early-passage iPSC-derived cultures (~30%) compared to ESC-derived cultures (~85.5%), which was in accordance with our immunocytochemistry observations (Figure 5B). This percentage increased to approximately 50% in early-passage iPSC neural induction day 15 cultures (data not shown). Consistent with the PCR analysis, the late-passage iPSCs at neural induction day 7 contained a comparable percentage of CD24^+ ^cells when compared to ESCs (~83%, Figure 5B). Together, these results showed that extended passaging enhances iPSC homogeneity and similarity to ESCs in our culture system.

### iPSC derived neurons exhibit an improved functional profile after extended passaging

**Figure 6:**
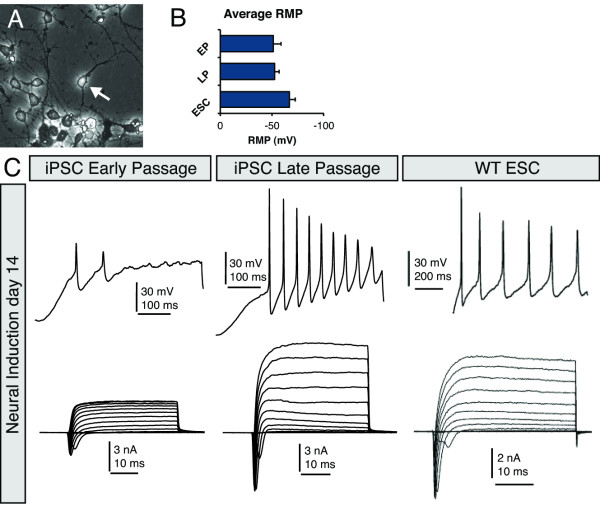
**Electrophysiological assessment of GG3.1 iPSC-derived neurons**. (**A**) Micrograph showing a representative neuronal cell that was targeted for recording. (**B**) Average resting membrane potentials for one set of experiments shows no discernable difference between EP and LP neurons. (**C**) Representative traces for whole cell-patch clamp recordings in EP, LP and WT ESCs. Action potentials were elicited with 500 ms long current injections of 2-340 pA. Current recordings were generated by stepping up membrane potential from -90 mV to +60 mV in 10 mV increments.

To evaluate the functional status of iPSC-derived neurons, we performed whole cell patch clamp experiments between days 7-14 of neural induction (Figure 6). For consistent analysis, we chose cells with a distinct bipolar or multipolar morphology (Figure 6A). The average resting membrane potentials were similar between early and late-passage iPSCs at ~55 mV, which was more depolarized than those recorded in ESCs (Figure 6B). Using a current step protocol, ~90% of patched ESC-derived neurons elicited repeated action potentials and robust inward and outward currents (Figure 6C). By contrast, early-passage iPSC-derived neurons, although morphologically similar to ESC-derived cells, produced only solitary or paired action potentials with comparatively weak inward and outward currents (Figure 6C). Action potentials were recorded from only ~23% of cells. Hyperpolarizing the cells (to ~-70 mV) typically did not substantially enhance the ability of early-passage iPSC-derived neurons to generate repetitive action potentials. Moreover, these cells displayed poor membrane integrity, as indicated by low input resistances that tended to get even lower fairly rapidly, which made recording difficult. Late-passage iPSC-derived neurons were capable of producing action potentials of similar amplitude and frequency as ESC-derived neurons. Robust action potentials were recorded from ~58% of cells (Figure 6C). Accordingly, the inward and outward currents (most likely sodium and potassium currents, respectively, although this was not empirically determined) were equivalent with those detected in ESC neurons (Figure 6C).

## Discussion

To our knowledge, this is the first study to specifically compare the neural differentiation capacity between early- and late-passage murine iPSCs. Of our four iPSC lines, three (GG3.1/3 and miPS-25) generated neuronal populations greater than 30% (n = 3 per line) of the total cell populations in early-passage culture when we applied an ESC-based neuronal induction protocol. Our group and others have previously shown that this protocol yields neuronal population of greater than 80% purity using murine ESCs [29,33]. Quantitative gene expression analysis revealed a similar, but temporally delayed pattern of neural lineage gene expression between ESCs and one iPSC line (GG3.1). We found that serial passaging improved the stability and maintenance of two newly derived iPSC lines in an undifferentiated state (Figure 3). Moreover, upon neural induction, late-passage iPSCs and ESCs undergo nearly identical temporal changes in gene expression (Figures 4 and 5). These results strongly suggest that sufficient cellular divisions are necessary to generated stable iPSCs clones that can achieve directed differentiation efficiencies comparable to ESCs.

The increase in expression of pluripotency factors in late-passage GG3.1 cells (Figure 4B) seems to agree with previous reports showing that differential gene expression between ESCs and iPSCs diminishes after passaging [16,17]. Since the RNAs for our analyses were extracted from whole cell populations, we must be careful in drawing conclusions about the individual cells within iPSC populations. The qRT-PCR data in Figure 4B is more an indication of the homogeneity of undifferentiated cultures, than a direct measure of pluripotency. For instance, the mRNA from early-passage cultures may be diluted by the mRNA of spontaneously differentiated cells, which would lower the measured relative expression of genes uniquely expressed in undifferentiated cells. Thusly, these data suggest that late-passage GG3.1 cultures contain a pluripotent population of cells roughly as homogeneous as our ESC cultures. Alternatively, we can conclude that the pluripotent state of these iPSC lines is more stable at later passages. Likewise, our analyses of neural markers in Figure 5 demonstrates the comparatively equivalent percentage of cells expressing these genes in late-passage GG3.1 and ESC cultures at each time point. These similarities in gene expression are particularly noteworthy when one considers that GG3.1 iPSCs and ESCs were derived from mice with disparate genetic backgrounds (i.e. B6-CD1 and R1, respectively).

Our results also point to functional differences between early-passage and late-passage iPSC-derived neurons. However, it is important to note that the results in Figure 6 are not entirely comprehensive in their assessment of each neural induction culture. For instance, we did not label a specific subtype of neurons for analysis (e.g. glutamatergic or GABAergic neurons); thus, the neurons analyzed may have represented multiple phenotypes despite having a similar morphology. In future studies, the use of subtype-specific fluorescent reporters may allow for more precise assessment of a particular population of stem cell-derived neurons. Regardless of these technical limitation, the generation of repeated action potentials with corresponding Na^+^/K^+ ^currents was used as a general criterion for excitatory functional neurons. In early-passage cultures, we were unable to record repeated action potentials even after 14 days of differentiation. This indicates that neurons developing in early-passage cultures may be functionally defective. We speculate that the extreme heterogeneity of early-passage neural cultures may create an environment that is not conducive to functional maturation.

A growing body of work has demonstrated that iPSCs can give rise to a wide array of neural subtypes using protocols optimized for ESCs [9,12,38,39]. However, few studies consider thoroughly the relative efficiency with which differentiation occurs between ESCs and iPSCs. Recently, Hu et al. published work showing that human iPSC lines derived using disparate methods (i.e. integrating and non-integrating vectors) displayed variable efficiencies when directed to differentiate into motor neurons [38]. Remarkably, cell lines derived using non-integrating episomal expression of the transgenes appeared to be just as susceptible to variation in differentiation potency as cells derived using retroviruses, which suggests that variability is independent of derivation method. These findings are reminiscent of our initial comparison of early-passage iPSCs and ESCs in that differentiation potency failed to match that seen in ESCs. It is noteworthy that the passage numbers of the iPSC cell lines used by Hu et al. were not reported, so it is possible that these observed differences could be attenuated with sufficient cellular turnover. More recently, Boulting et al. found that early- and late-passage human iPSCs performed similarly during motor neuron differentiation and functional analysis, despite karyotypic abnormalities in some late-passage cell lines [39].

Since varying differentiation propensities among iPSC lines appear to be independent of derivation methods, the beneficial effect of repeated passaging may reveal an underlying feature of cellular reprogramming in general. It has been proposed that a residual signature or "memory" of the cell type of origin persists throughout the reprogramming process in the form of hypo- or hypermethylated regions of the genome and/or aberrant gene expression [26-28]. It is possible that hypermethylation of neural gene promoter regions may have confounded early-passage iPSC differentiation, although we did not directly test this. Several new studies also report the generation of genetic mutations, deletions and copy number variations during the reprogramming process [18,19,21]. Over successive cellular divisions, however, it appears that epigenetic marks are progressively "erased" or, perhaps, selected against. At the moment, the precise mechanisms of this process are unclear, but the epigenetic signature appears to be a phenomenon in both mouse and human reprogrammed cells [17,26,27]. Of note, Hussein and colleagues recently demonstrated that early-passage human iPSC lines have a high prevalence of genetic copy number variations. Surprisingly, the amount of copy number variations declined rapidly over successive passages (i.e. > 15 passages) seemingly due to selective pressure on the aberrant cells [19]. It is feasible that this phenomenon is reflected in our current observations. For future investigations it will be necessary to examine karyotypic stability and copy number variation over the course of these experiments to determine if neural differentiation is impacted by these factors.

## Conclusions

The work presented herein demonstrates that extended passaging can lead to more stable iPSCs, which in turn leads to more efficient neural differentiation. The utility of this approach will certainly be elucidated by further studies examining the effect of passaging on chromosomal stability in iPSCs. Importantly, the present results highlight the need for improved screening methodologies to isolate iPSC clones with the greatest potential for directed differentiation. Future studies identifying methylation signatures that define fully reprogrammed iPSCs will be helpful in developing better assays to evaluate the progression of reprogramming. Interestingly, some reports suggest that neuronal conversion of recalcitrant iPSCs can be greatly improved through treatment with chromatin-modifying drugs or small molecules [27,39,40]. Undoubtedly, for the eventual application of iPSCs in disease modeling or cell replacement therapies, complete reprogramming will be critical for unbiased analysis of disease progression and safety.

## Methods

### ES and iPS cell culture, maintenance and analysis

iPSCs were generated by transducing mouse embryonic fibroblasts (for genetic backgrounds see Supplementary Table 1 in Additional File 1) with Moloney murine leukemia viruses (MMLVs) carrying the coding regions of mouse *Oct4, Sox2, Klf4 *and/or *Nanog *or human *Oct4, Sox2 *and *Klf4*. R1 mouse embryonic stem cells and iPSCs were maintained in culture as described previously (Figure 1A) [29]. Briefly, iPS and ES cells were plated on gelatin-coated tissue culture plates and grown in high-glucose Dulbecco's Modified Eagle's Medium (DMEM) (Invitrogen) supplemented with 15% FBS (Invitrogen), 1.0 mM sodium pyruvate (Stemcell Technologies), 10 mM nonessential amino acids (Stemcell Technologies), 0.01% penicillin streptomycin (Stemcell Technologies), 2.0 mM L-glutamine (Stemcell Technologies), 1,000 units/ml leukemia inhibiting factor (Chemicon), and 0.055 mM 2-mercaptoethanol. Cells were passaged by dissociation with 0.25% trypsin-EDTA every 2-3 days. Two days after passaging the health and phenotypic stability of the cells was assessed. Five to ten representative DIC images were taken and then analyzed on MetaMorph software. Dissociation of tightly packed clones and/or the appearance of enlarged and flattened cells were indicators of spontaneous differentiation.

### Neural induction

After 6-8 (early) and 20-30 (late) passages, iPSC and late-passage (30-40) ESCs were subjected to neural differentiation according to a previously established procedure for ESCs (Figure 1A) [29,33]. Cells were dissociated into single cells using 0.25% trypsin-EDTA and resuspended in differentiation medium containing Glasgow's Minimum Essential Medium (GMEM) (GIBCO/Invitrogen), 5% Knockout serum replacement (Invitrogen), 2.0 mM L-glutamine, 1.0 mM sodium pyruvate, 0.1 mM nonessential amino acids, 0.01% penicillin streptomycin, and 0.1 mM 2-mercaptoethanol. Cells were plated on gelatin-coated plates for 40 minutes to remove any residual feeder cells or partially differentiated cells. Cells were then cultured in low adherence 100 mm bacterial plates for 5 days at a density of 5-10 × 10^4 ^(iPSC) or 5 × 10^4 ^(ESC) cells per ml to allow embryoid body (EB) formation. Differentiation medium was changed at day 3. On day 5, EBs were plated *en bloc *on tissue culture plates or chamber slides double-coated with poly-D-lysine (200 μg/ml) and mouse laminin (10 μg/ml) at a density of 1-2 × 10^2 ^EBs per cm^2 ^in fresh medium. Before plating, EB were imaged to assess size and shape. At least 50 EBs were analyzed using MetaMorph software to determine the average EB diameter for each biological replicate. Twenty-four-thirty-six hours post plating, the medium was changed to neural induction medium containing GMEM, 1% N2, 2 mM glutamine, 1 mM sodium pyruvate, 0.1 mM nonessential amino acids, 0.1 mM 2-mercaptoethanol, 0.01% penicillin streptomycin and 10 ng/ml brain-derived neurotrophic factor (BDNF) (PeproTech). Neural induction cultures were maintained for 3, 7 or 15 days before cells were harvested for RNA extraction, electrophysiological recordings, flow cytometry analysis, or fixation with 4% paraformaldehyde for immunocytochemistry.

### Quantitative RT-PCR

The relative expression levels of pluripotency markers and early/mature neural markers were assessed by conventional reverse transcriptase PCR (RT-PCR) or quantitative real-time RT-PCR (qRT-PCR) using a previously described procedure [41]. At various time points of cell culture and neural induction (undifferentiated day 5-7, EB day 5, and days 3, 7 and 15 of neural induction), total RNA was isolated using the RNeasy Minikit (Qiagen) and then treated with TURBO DNase (Ambion) to decrease the likelihood of DNA contamination. Single-stranded cDNA was synthesized using Omniscript reverse transcriptase (Qiagen) and Oligo-dT primers. All amplicons had standardized sizes of 100-110 bps. For non-quantitative RT-PCR, the resultant cDNA was amplified with Platinum Taq DNA polymerase (Invitrogen) for 30 cycles. For qRT-PCR, the cDNA samples were amplified on an ABI PRISM 7900HT Sequence Detection System (Applied Biosystems) using the SYBR Green PCR Master Mix (Applied Biosystems). For each PCR reaction, a mixture containing cDNA template (5 ng), Master Mix, and forward and reverse primers (400 nM each) was treated with uracil N-glycosylase at 50°C for 2 min before undergoing the following program: 1 cycles, 95°C, 10 min; 45 cycles, 95°C, 15 sec, 60°C, 1 min; 1 cycles, 95°C, 15 sec, 60°C, 15 sec, 95°C, 15 sec (for melting curve analysis); 72°C, hold. Melting curve analysis was performed to confirm the authenticity of the PCR products. For internal control, PCR was run with cDNA samples using an L27 (ribosomal housekeeping gene) primer pair, whose PCR product crosses an intron. To check the efficiency of primer pairs, a cDNA dilution series (1, 1/10, 1/100, and 1/1,000) was amplified. The mRNA level for each gene was calculated relative to L27 mRNA expression. L27 expression was previously determined to be stable under all experimental conditions [29]. Each data point represents the average of 7-10 replicates from 3-4 biological samples. Statistical significance was determined using a One-Way ANOVA followed by Scheffe's *post-hoc *test. Primer sequences used in this study are listed in *Supplementary Table 2* (Additional File 1).

### Immunocytochemistry

Prior to differentiation and at days 3 and 7 of neural differentiation, cultures were fixed with 4% paraformaldehyde for 30 min. Chamber slides were incubated in blocking solution and then with a primary polyclonal and a monoclonal antibody together. Primary antibodies used in this study are listed in *Supplementary Table 3* (Additional File 1). Immunoreactivity with monoclonal and polyclonal antibodies was visualized by using an Alexa Fluor 488 conjugated anti-mouse IgG and Alexa Fluor 568 conjugated anti-rabbit IgG, respectively. For visualizing cellular nuclei, the specimens were counterstained with DAPI (Vector, VectaShield). Expression of certain proteins was quantified using the imageJ (NIH) cell counting plug-in. Regions with moderate cellular densities were chosen at random for 3 biological samples unless stated otherwise.

### Electrophysiology

Whole cell patch-clamp recordings were conducted as described previously [29]. Briefly, experiments were performed using an EPC-10 amplifier, and data was acquired using the Pulse program (HEKA Electronics). Putative bipolar neurons were selected for recording based on morphology. The pipette solution contained: 140 mM KCl, 5 mM MgCl2, 5 mM EGTA, 2.5 mM CaCl2, 4 mM ATP, 0.3 mM GTP, and 10 mM Hepes, pH 7.3 (adjusted with KOH). The bathing solution contained: 140 mM NaCl, 1 mM MgCl2, 5 mM KCl, 2 mM CaCl2, 10 mM Hepes, and 10 mM glucose, pH 7.3 (adjusted with NaOH). Voltage-clamp and current-clamp data was analyzed using the Pulsefit (HEKA Electronics), Origin (OriginLab) and Microsoft Excel software.

### Flow cytometry

Cells were dissociated by a brief exposure to 0.25% trypsin-EDTA. After blocking with serum, cells were incubated with one of the following primary antibodies: anti-CD24-phycoerythrin (PE), mouse immunoglobulin G (IgG) isotype control or Alexa 568-conjugated anti-rabbit secondary antibody. Cell sorting and analysis were performed with a FACSCalibur flow cytometry system (BD Biosciences). Data analysis was performed using FlowJo 8.6.6 software (Tree Star, Inc.).

## Authors' contributions

KRK designed the experiments and performed the cell culture, immunocytochemistry, flow cytometry and qPCR analysis and drafted the manuscript. PT and SV generated the iPSC lines. TK assisted with cell culture and data analysis. JWT and TRC conducted the electrophysiological analyses. EH aided in the conceptual design of the study, monitored the experiments and helped draft the manuscript. All authors read and approved the final manuscript.

## Supplementary Material

Additional File 1 **Supplementary information**. Supplementary Figures 1-3 and Tables 1-3. **Supplementary **Figure 1 **- iPSCs (GG3.3 and miPS-20) at various stages of neural differentiation**. Representative micrographs of miPS-20 (**A**) and GG3.3 (**B**) iPSCs prior to differentiation, on day 5 of EB formation and on days 3 and 7 of neural induction. (**C-E**) Examples of aberrant cell types with endodermal (**C**) and mesodermal (**D, E**) morphologies that were prevalent during all early-passage iPSC Ni experiments. Scale bars represent 100 μm. **Supplementary **Figure 2 **- The GG3.1 cell line is a competent iPSC line with no detectable transgene re-expression during neural differentiation**. (**A**) Alkaline phosphatase staining of ESC and GG3.1 cells indicates pluripotent cells in undifferentiated cultures and a gradual loss of pluripotency during the EB stage. (**B**) Primers amplifying an untranslated region (UTR) of the Oct4, Sox2 and Klf4 genes were compared to exon expression in undifferentiated and neural induction days 3 and 7. All expression levels were normalized to undifferentiated expression levels. The identical pattern of expression indicates a lack of transgene re-expression. (**C-D**) The GG3.1 cell line displays similar expression levels of the *Dlk1-Dio3 *locus genes Gtl2 and Rian, which is an indirect measure of complete reprogramming. Equivalent expression of these genes was validated using 2 different primer sets; one novel and one published by Stadtfeld et al., 2010. Values are mean ± SD for 2-3 independent samples. **Supplementary **Figure 3 **- Expression of neural lineage and subtype specific genes throughout Ni of early-passage GG3.1 iPSCs**. (**A-C**) Representative micrographs showing the presence and abundance of HuC/D, Map2, neurofilament (NF) and Calretinin (Calr) positive cells at Ni day 7. Scale bars represent 150 μm. (**D**) The pluripotency marker Rex1 is downregulated during differentiation. (**E**) The anterior neurodevelopmental gene Otx2 is expressed by day 5 of EB. The neurotrophin receptor TrkB is expressed during the EB stage, but expression is elevated by day 7 of Ni. Calretinin is expressed by Ni day 3. (**F**) Markers of glutamatergic neurons, vesicular glutamate transporter 2 (VGLUT2) and the AMPA receptor subunit GluR2 are highly expressed by days 7 and 15 of Ni. Likewise, the GABAergic neuronal marker, glutamic acid decarboxylase 1 (GAD1) is unregulated by days 7 and 15. Values are mean ± SD for 2-3 independent samples. **Supplementary Table 1 - Pluripotent stem cell lInes. Supplementary Table 2 - Primers. Supplementary Table 3 - Antibodies**. Click here for file
