# Supplementary material for: Extended passaging increases the efficiency of neural differentiation from induced pluripotent stem cells
Source: BMC Neurosci. 2011 Aug 10;12:82. doi: 10.1186/1471-2202-12-82 (PMC3167757; doi:10.1186/1471-2202-12-82)
Supplement: Additional File 1 — Supplementary information. Supplementary Figures 1-3 and Tables 1-3. Supplementary Figure 1 - iPSCs (GG3.3 and miPS-20) at various stages of neural differentiation. Representative micrographs of miPS-20 (A) and GG3.3 (B) iPSCs prior to differentiation, on day 5 of EB formation and on days 3 and 7 of neural induction. (C-E) Examples of aberrant cell types with endodermal (C) and mesodermal (D, E) morphologies that were prevalent during all early-passage iPSC Ni experiments. Scale bars represent 100 μm. Supplementary Figure 2 - The GG3.1 cell line is a competent iPSC line with no detectable transgene re-expression during neural differentiation. (A) Alkaline phosphatase staining of ESC and GG3.1 cells indicates pluripotent cells in undifferentiated cultures and a gradual loss of pluripotency during the EB stage. (B) Primers amplifying an untranslated region (UTR) of the Oct4, Sox2 and Klf4 genes were compared to exon expression in undifferentiated and neural induction days 3 and 7. All expression levels were normalized to undifferentiated expression levels. The identical pattern of expression indicates a lack of transgene re-expression. (C-D) The GG3.1 cell line displays similar expression levels of the Dlk1-Dio3 locus genes Gtl2 and Rian, which is an indirect measure of complete reprogramming. Equivalent expression of these genes was validated using 2 different primer sets; one novel and one published by Stadtfeld et al., 2010. Values are mean ± SD for 2-3 independent samples. Supplementary Figure 3 - Expression of neural lineage and subtype specific genes throughout Ni of early-passage GG3.1 iPSCs. (A-C) Representative micrographs showing the presence and abundance of HuC/D, Map2, neurofilament (NF) and Calretinin (Calr) positive cells at Ni day 7. Scale bars represent 150 μm. (D) The pluripotency marker Rex1 is downregulated during differentiation. (E) The anterior neurodevelopmental gene Otx2 is expressed by day 5 of EB. The neurotrophin receptor TrkB is expressed [file 1471-2202-12-82-S1.PDF]

Supplementary Figures and Tables

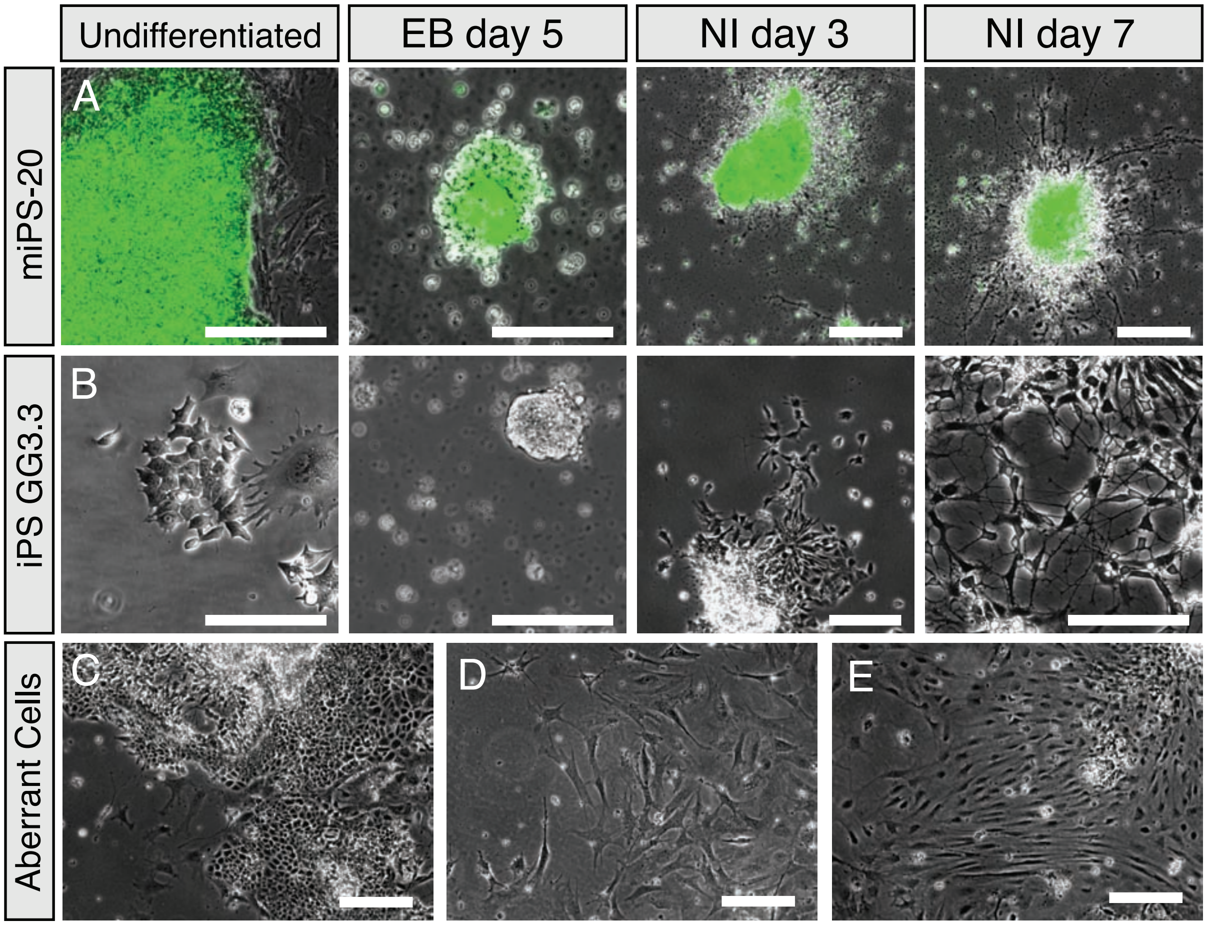

Supplementary Figure 1 - iPSCs (GG3.3 and miPS-20) at various stages of neural differentiation. Representative micrographs of miPS-20 (A) and GG3.3 (B) iPSCs prior to differentiation, on day 5 of EB formation and on days 3 and 7 of neural induction. (C-E) Examples of aberrant cell types with endodermal (C) and mesodermal (D, E) morphologies that were prevalent during all early-passage iPSC Ni experiments. Scale bars represent 100 μm.

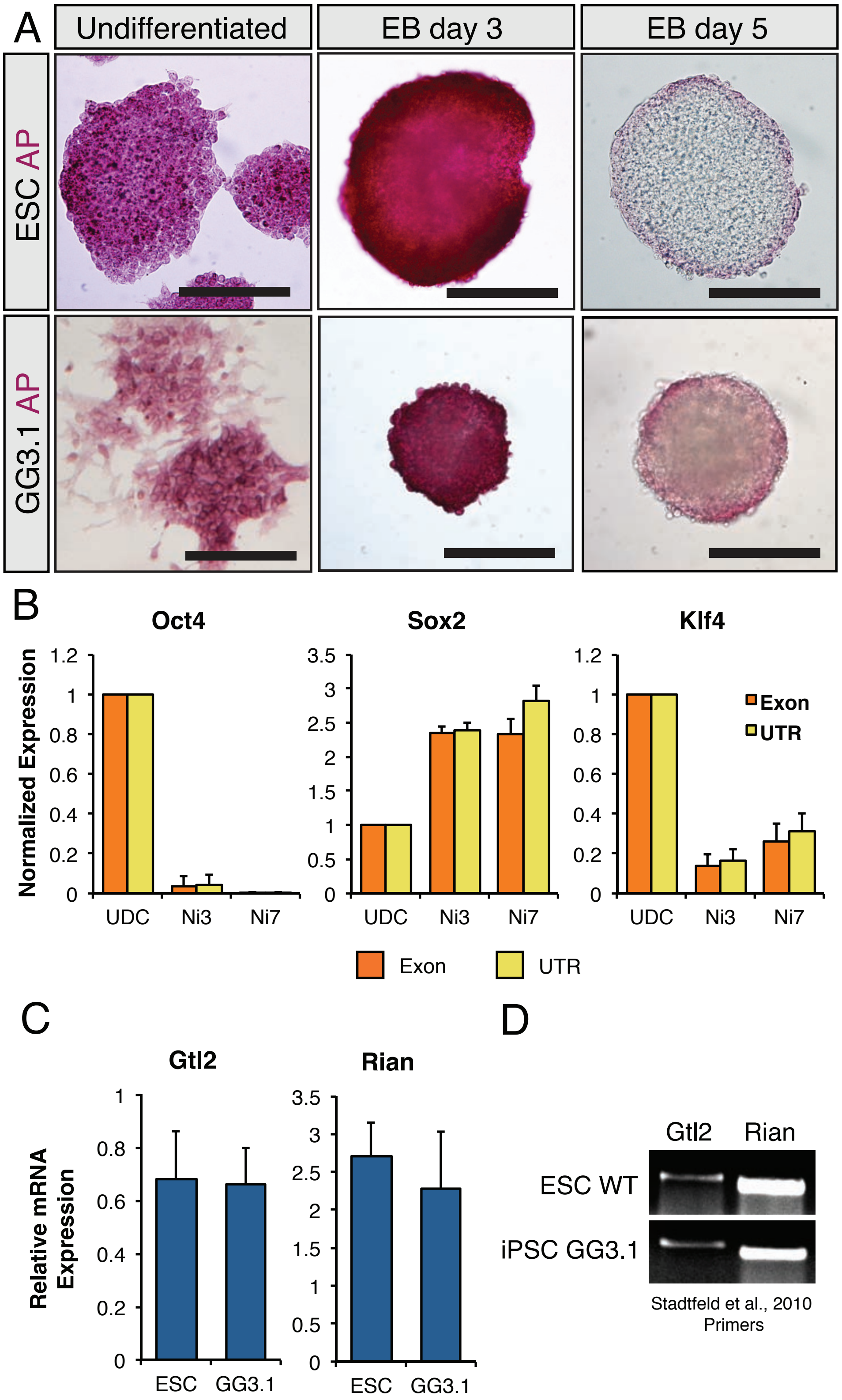

Supplementary Figure 2 - The GG3.1 cell line is a competent iPSC line with no detectable transgene re-expression during neural differentiation. (A) Alkaline phosphatase staining of ESC and GG3.1 cells indicates pluripotent cells in undifferentiated cultures and a gradual loss of pluripotency during the EB stage. (B) Primers amplifying an untranslated region (UTR) of the Oct4, Sox2 and Klf4 genes were compared to exon expression in undifferentiated and neural

induction days 3 and 7. All expression levels were normalized to undifferentiated expression levels. The identical pattern of expression indicates a lack of transgene re-expression. (C-D) The GG3.1 cell line displays similar expression levels of the Dlk1-Dio3 locus genes Gtl2 and Rian, which is an indirect measure of complete reprogramming. Equivalent expression of these genes was validated using 2 different primer sets; one novel and one published by Stadtfeld et al., 2010. Values are mean ± SD for 2-3 independent samples.

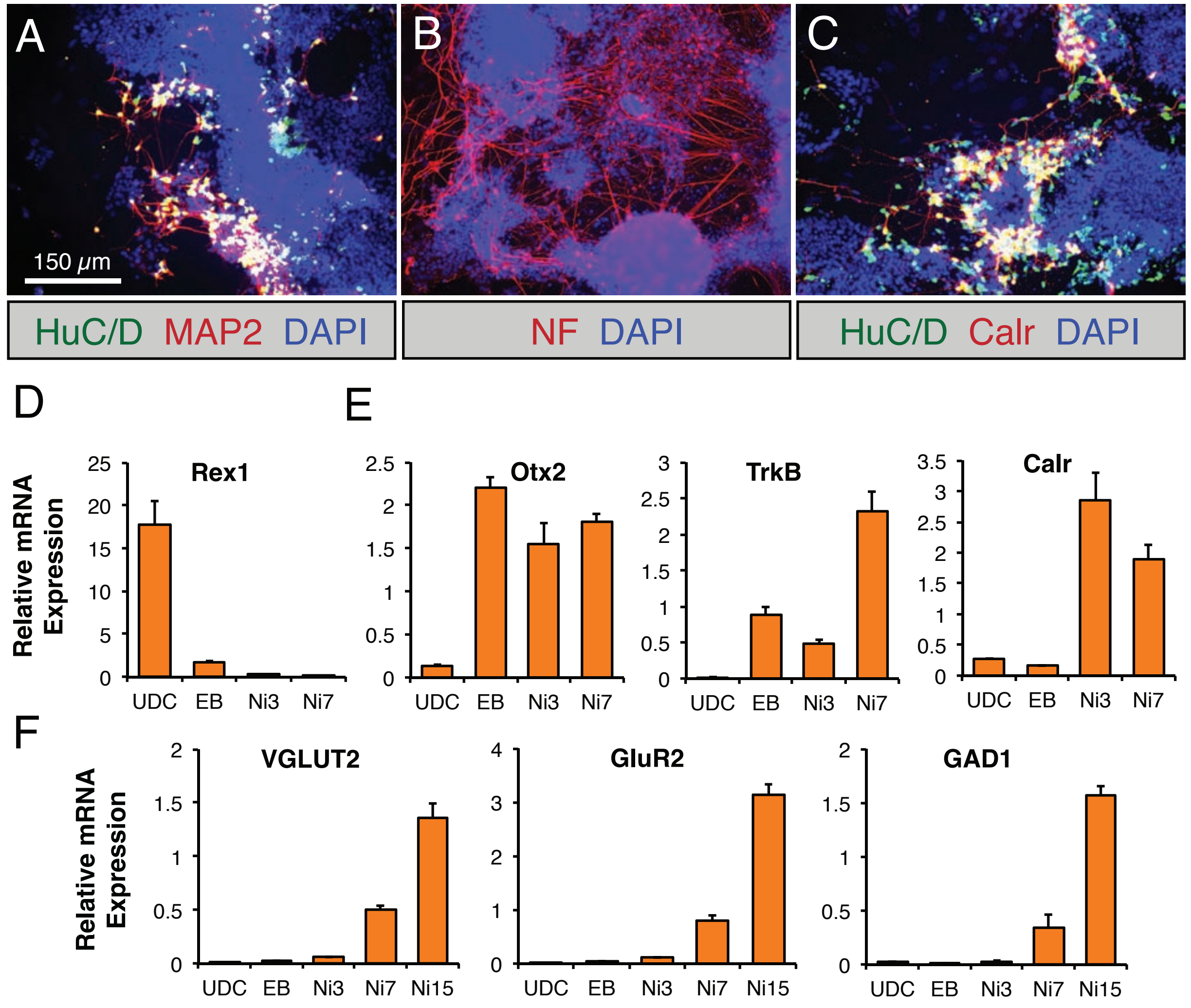

Supplementary Figure 3 - Expression of neural lineage and subtype specific genes throughout Ni of early-passage GG3.1 iPSCs. (A-C) Representative micrographs showing the presence and abundance of HuC/D, Map2, neurofilament (NF) and Calr positive cells at Ni day 7. Scale bars represent 150 μm. (D) The pluripotency marker Rex1 is downregulated during differentiation. (E) The anterior neurodevelopmental gene Otx2 is expressed by day 5 of EB. The neurotrophin receptor TrkB is expressed during the EB stage, but expression is elevated by day 7 of Ni. Calretenin (Calr) is expressed by Ni day 3. (F) Markers of glutamatergic neurons, vesicular glutamate transporter 2 (VGLUT2) and the AMPA receptor subunit GluR2 are highly expressed by days 7 and 15 of Ni. Likewise, the GABAergic neuronal marker, glutamic acid decarboxylase 1 (GAD1) is unregulated by days 7 and 15. Values are mean ± SD for 2-3 independent samples.

Supplementary Table 1: Pluripotent Stem Cell Lines

Name	Genetic Background	Source Tissue	Factors	Method
miPS-20	CD1-Oct4GFP	embryonic fibroblast	mouse	Retrovirus
miPS-25	CD1-Oct4GFP	embryonic fibroblast	mouse +nanog	Retrovirus
GG3-1	B6-CD1	embryonic fibroblast	human	Retrovirus
GG3-3	B6-CD1	embryonic fibroblast	human	Retrovirus
ESC	R1	blastocyst/inner cell mass	N/A	N/A

Supplementary Table 2: Primer Pairs

Gene Name	NCBI mRNA acc. #	Forward	Reverse
BMP4	NM_007554.2	GAGGAGGAGGAAGAGCAGAG	TGGGATGTTCTCCAGATGTT
Calretinin	NM_009788.4	GGAGATGAACATCCAACAGC	TCACTGCAGAGCACAATCTC
GAD1	NM_008077.4	TTCTGGTACATTCCACAAAGC	CCTGACTCCATCATCAGAGC
GluR2	NM_001039195.1	CTGGTGGCTTTGATTGAGTT	AATTCTGCGAGGAAGATGG
HuC/D	NM_010487.2	GAGAGGAGAGGACCCATCAC	GCCTGAAGAAGTCGCTCTTT
Musashi1	NM_008629.1	CGTCACTTTCATGGACCAG	CTTAGGCTGTGCTCTTCGAG
Nanog	NM_028016.2	AACCAAAGGATGAAGTGCAA	CAATGGATGCTGGGATACTC
Neurogenin1	NM_010896.2	TCGGCTTCAGAAGACTTCAC	GTGGTATGGGATGAAACAGG
NSE	NM_013509.2	AACGTCGGCATCCAGATAGT	GATTGACCTTGAGCAGCAAA
Oct4	NM_013633.2	AAGCCGACAACAATGAGAAC	CTCCAGACTCCACCTCACAC
Otx2	NM_144841.3	AGAATCCAGGGTGCAGGTAT	CAGGCCTCACTTTGTTCTGA
Rex1	NM_009556.3	GGGTACGAGTGGCAGTTTCT	CGTGTCCCAGCTCTTAGTCC
Sox1	NM_009233.3	AACCAGGATCGGGTCAAG	ATCTCCGAGTTGTGCATCTT
Sox2	NM_011443.3	GCTCGCAGACCTACATGAAC	CCTCGGACTTGACCACAGA
Synaptophysin	NM_009305.2	GACGAGGTGAAAGCTGAGAC	CAGAGAGGGCTGTCTAGGG
Tau	NM_001038609.1	TAGCAACGTCCAGTCCAAGT	GTCACTTTGCTCAGGTCCAC
VGlut2	NM_080853.3	ACCCAGATTCCAGGAGGATA	GCAGATGGGATCAGCATATT
Klf4	NM_010637.3	TTGTGACTATGCAGGCTGTG	CCCAGTCACAGTGGTAAGGT
Sox2-UTR	NM_011443.3	AGGAAGGAGTTTATTCGGATTT	GCCTTAAACAAGACCACGAA
Oct4-UTR	NM_013633.2	AAGAGAACCTGGAGCTTTGG	CAACAGCATCACTGAGCTTC
Klf4-UTR	NM_010637.3	GGAAGCGATTCAGGTACAGA	ATGCAGCAGTTGGAGAACTT
Gtl2	NR_027652.1	TTTGTAGAGCCAAGGAGCAC	ATGATCCTCTTTGTCGCTTG
Rian	NR_028261.1	CGAGACACAAGAGGACTGCT	GTCCAGTTGCCCTCTCAGTA

Supplementary Table 3: Antibodies

Name	Isotype	Dilution (1:X)	Supplier	Cat#
Sox2	Rabbit IgG	333	Abcam	ab59776
TuJ1	Mouse IgG	500	Covance	MMS-435P
MAP2	Rabbit IgG	1000	Chemicon	AB5622
HuC/D	Mouse IgG	50	Molecular Probes	A21271
Brn3a	Mouse IgG	500	Chemicon	MAB1585
Synaptophysin	Rabbit IgG	500	Zymed	18-0130
NeuN	Mouse IgG	500	Chemicon	MAB377
Neurofiliment	Rabbit IgG	1000	Chemicon	AB1983
CD24-PE	Mouse IgG	1 ug/1e6 cells	Abcam	ab25494
